# Breaking barriers, building futures: Inclusive workplaces for autistic people

**DOI:** 10.3389/fpsyt.2025.1606075

**Published:** 2025-11-11

**Authors:** Chiara Failla, Paola Chilà, Dario Baluci, Carmelo Caporlingua, Anna Meduri, Giovanni Pioggia, Flavia Marino

**Affiliations:** 1 Institute for Biomedical Research and Innovation (IRIB), National Research Council of Italy (CNR), Messina, Italy; 2 Faculty of Psychology, International Telematic University Uninettuno, Roma, Italy; 3 Società Cooperativa Sociale Audacia, Messina, Italy; 4 Department of Cognitive, Psychological Science and Cultural Studies, University of, Messina, Italy

**Keywords:** autism, work, inclusion, Quality of Life, workplace

## 1 Introduction

The employment of autistic person is a highly relevant social issue that deserves attention from society. Although in Europe it is estimated that around 5 million people, or 0.67% of the population, are on the autism spectrum, employment rates remain low: only 10% compared to 47% of people with other disabilities (1). Wanting to explain the characteristics of autism, we want to remember that we are referring to a neurodevelopmental condition characterized by difficulties in social interactions and restricted and repetitive behaviors (2) and that its manifestation is highly heterogeneous and this underlines the importance of considering each individual as a unique person with distinct abilities and characteristics (3). Approximately 50% of autistic people have an average or above-average IQ and have no significant physical needs, demonstrating that the vast majority of these individuals can and want to work (4). In the literature, it is possible to find bibliographic references that highlight the strengths of autistic people, such as high concentration, attention to detail, recognition of systematic patterns and efficiency in carrying out routine tasks (4). These qualities allow them to excel in technological fields such as coding and software creation, minimizing errors. Other distinctive characteristics that include a high degree of reliability, confidence and patience for repetitive tasks tend to make them particularly suitable for monotonous, repetitive jobs that require little social interaction (5). Some individuals on the autism spectrum may be suited to tasks that rely on these characteristics however, such claims should always be considered on an individual level to avoid stereotypes. Despite these skills, employers often have prejudices, associating the employment of autistic individuals with higher supervision costs and presumed reduction in productivity (6). Among the obstacles to hiring autistic individual, traditional recruitment methods that emphasize social skills certainly represent another obstacle. This approach contributes to perpetuating stereotypes and discrimination, limiting their access to the labor market (7). Employment and therefore work responsibility play a central role in the lives of adults as they guarantee financial independence and above all contribute to improving self-esteem and self-perception in terms of self-efficacy (8). For autistic people, work represents a means to achieve a better quality of life and an opportunity to show their capabilities. Investing resources in this area can certainly favor a path of greater social inclusion and better general well-being for autistic person. Advances in digital technologies can offer promising solutions to overcome the challenges faced by autistic people the workplace. In this context, technological innovations are not only tools to break down barriers to inclusion, but also a strategic lever to improve the capabilities of autistic individuals. The development of personalized assistive technologies, such as stress monitoring devices or intelligent work systems that dynamically adjust light, noise and temperature (9) can be key elements in favoring effective work integration. The inclusion of autistic person represents both a challenge and an extraordinary opportunity for society and the economy (10). New technologies can represent a strategic lever to facilitate job placement, through personalized solutions and more accessible work environments (9, 10). Promoting the inclusion of people with ASD is not only an ethical challenge, but also an opportunity to enhance often invisible talents. In this article, we focus primarily on experiences and interventions for adults on the autism spectrum who require mild to moderate levels of support (terminology used in line with the DSM-5), given that most documented employment programs address this group, recognizing that the autism spectrum is extremely heterogeneous and that support needs vary widely.

## 2 Technological innovation and employment inclusion

Digital technologies (DT) have revolutionized the labor market in several sectors and have opened up the possibility of improving work inclusion for autistic people (11). An effective solution for those with social difficulties is represented by the use of tools such as email, chatbots and digital communication platforms thus reducing the stress associated with face-to-face interactions and creating a more accessible environment (12). Another clear advantage of DT is certainly the possibility of allowing flexible working arrangements, ideal for meeting the sensory and cognitive needs of those who have difficulty coping with traditional work environments (13). An example in the scientific literature of technology for inclusion is the “Roim Rachok” program in Israel, which trains young autistic individuals as aerial photography interpreters. This program leverages the attention to detail and visual analysis skills characteristic of many individuals on the autism spectrum, providing them with meaningful roles in highly structured and technology-supported work environments. Through measurements with the QoL-Q scale, a significant improvement in the quality of life of participants with DSA was recorded after six months of operational work in the military unit in the areas of personal satisfaction, professional competence and independence. This change occurred both during training but especially after the actual work integration in an inclusive and structured environment. These results demonstrate how real work experience can actually have a positive impact on perceived well-being (14). Similarly, assistive technologies (AT) represent a resource to address the sensory and stress management difficulties experienced by autistic people. Sensor-equipped bracelets, as well as other wearable devices capable of monitoring stress, are useful tools to provide real-time feedback and support the creation of personalized interventions for better emotional management (9). The focus on creating more comfortable and therefore potentially more productive spaces for autistic individuals (12), corresponds to a further effective element for greater work integration and in fact integrated systems such as intelligent work environments, capable of dynamically regulating parameters such as lighting, temperature and noise can be facilitators for achieving adequate performance (15). Another crucial aspect can be performed by mobile applications and software for time management and planning that help reduce organizational difficulties, allowing more efficient management of complex activities (16). Virtual reality (VR) in this context represents an innovative opportunity, Allowing the simulation of work environments to prepare autistic individuals for real interviews and professional situations (17–19).

## 3 Valuing neurodiversity in the digital economy

Autistic individuals possess unique characteristics that make them particularly suited to technological sectors (20). Work environments dedicated to data analysis, programming and other sectors characterized by attention to detail and importance in recognizing patterns with a high resistance to repetitive tasks are a fertile work environment for autistic individuals (4). These skills have been recognized by companies such as SAP and Microsoft, which have implemented specific programs, such as “Autism at Work”, to integrate autistic workers. These initiatives not only promote inclusion, but also contribute to innovation and company productivity, demonstrating that neurodiversity is a strategic factor for organizational success (6, 21–23). However, inclusion does not end with hiring. Autistic workers still face significant difficulties in integration, often due to stereotypes, communication barriers, and environmental sensitivities (24). In particular, there is a persistent misconception that autistic people lack social skills, which negatively impacts employment and career opportunities. In this context, selection tools such as structured interviews and competency-oriented assessments have proven to be fairer, allowing candidates to demonstrate their value beyond traditional social expectations (4, 11). Assistive technologies (AT) offer a concrete opportunity to overcome many of these barriers. Wearable devices capable of monitoring physiological parameters such as heart rate variability (HRV) or galvanic skin response (GSR) can provide real-time biofeedback, helping autistic people to manage emotional self-regulation, especially in intense or overstimulating work environments (9, 13). These tools become even more effective when integrated into smart environments capable of dynamically adapting to the individual’s sensory needs, modulating stimuli such as light, sound and temperature (15). In addition, digital applications for task management, shared calendars and planning systems support executive functions, facilitating the organization of complex or tight-term tasks (16). Based on the diagnostic criteria of the DSM-5, we propose a table (**Table 1**) that integrates the peculiarities of the autism spectrum with the available technological support, highlighting how certain tools can facilitate work inclusion. From this analysis, different types of assistive technologies emerge that can offer valid support in the professional environment.

**Table 1:** Mapping ASD challenges and digital interventions.

Code	Description	Observed challenges	Technological solutions
A.2 - Deficit in non-verbal communication	Limited use of gestures, facial expressions, or eye contact	Text-based chats/emojis/digital assistants: reduce the pressure of face-to-face communication	- Written communication or icon-based (e.g., digital PECS software)- Virtual assistants with avatars
A.3 - Difficulty in social relationships	Difficulty adapting to contexts, understanding implicit rules	Digital platforms with social scripts: provide examples and routines	- Bots simulating social scenarios- Social training apps with feedback
B.4 - Hyper/Hypo-reactivity to sensory input	Excessive or reduced reactions to sounds, lights, tactile stimuli	Wearable devices for monitoring and regulating sensory input	- Smartwatches detecting stress via HRV (e.g., Fitbit)- Apps with alerts for environmental stimuli (light/sound)
B.4 - Atypical interest in sensory aspects	Intense seeking of specific sensory stimuli (light, vibration, smell)	Customizable sensory wearables: provide regulated stimulation	- EEG/EMG headbands (e.g., Muse)- Devices with programmable stimuli for therapeutic use

Recent scientific literature has also highlighted the usefulness of asynchronous communication tools, such as emails, in reducing social stress in the most critical moments of working life – from recruitment to onboarding – offering autistic workers the opportunity to reflect before responding, reducing the anxiety related to immediate interaction (25). Although most of the studies on these technologies are still in the experimental phase and little applied to real work contexts, the importance of adopting a co-design approach, which directly involves autistic people in the development and validation of technological solutions, thus ensuring effective adherence to their needs, clearly emerges (9). Alongside technological solutions, it is essential to invest in the training of managers and colleagues through workshops conducted by neurodiversity professionals. These interventions contribute to dispelling widespread prejudices and to promoting a more empathetic and informed corporate culture (26, 27) Initiatives of this type should not be sporadic, but an integral part of broad and structured corporate strategies, aimed at enhancing neurodiversity as a resource for organizational resilience and competitiveness (26). Promoting the work inclusion of autistic people requires a multilevel approach, which combines the enhancement of individual strengths, the removal of systemic and interpersonal barriers, and the adoption of enabling technologies. When these components are strategically aligned, neurodiversity not only represents an ethical and social objective, but becomes an authentic driver of innovation and transformation for organizations (14).

## 4 Discussion

Promoting the work inclusion of autistic people in today’s digital society requires both a focus on the possibility of offering job opportunities and, above all, a reshaping of environments in which neurodivergent characteristics are welcomed as resources. Assistive technologies can play a central role in enhancing specific skills and reducing barriers related to communication and sensory regulation. The effectiveness of such tools depends on how much they reflect the real needs and preferences of their recipients. This underlines the importance of adopting co-design strategies that actively involve autistic people in the design and validation of possible technological solutions and alternatives to be developed. The main goal must be greater usability and acceptance, so we must aim from user-centered to user-driven innovation, also promoting empowerment and autonomy. In the (A)MICO project developed by Dei et al. (2023) (28), a co-design process involving five people that need minimal support to work was used to define the most appropriate feedback configuration. This approach allowed for the system to be analyzed from different perspectives and increased acceptance of the final product. Other recent studies (29, 30) have documented the effectiveness of co-design sessions with young adults with autism to better support life planning and independence to promote success for long-term employability. It is also necessary to aim for structured, accessible and sensorially adaptable work environments to promote greater effectiveness and work autonomy. All this must finally be integrated with adequate training of workers - managers, recruiters and human resources professionals - to challenge stereotypes, promote inclusive practices and build an informed corporate culture (31–33). These reflections are grounded in a synthesis of recent scientific literature on autism and employment inclusion, combined with documented examples of technological and organizational initiatives such as SAP’s and Microsoft’s “Autism at Work” programs and the clinical and research experience of the authors in the field of neurodevelopmental disorders. This integrated perspective aims to connect empirical evidence, applied practice, and policy directions to strengthen the reliability of the proposed framework. The synergistic integration of inclusive design, accessible environments, assistive technologies and organizational awareness can fully develop the potential of autistic people, transforming neurodiversity into a real strategic advantage for innovation, productivity and organizational resilience. A final but important consideration should be made in relation to the current rapid evolution of digital and assistive technologies; indeed, the framework proposed in **Table 1** is useful to consider in a dynamic and updatable manner. The increasing integration of artificial intelligence through adaptive systems, multimodal emotional recognition, predictive analytics, and personalized feedback has the potential to significantly improve the effectiveness and relevance of interventions (34). Real-time systems based on IoT and deep learning have achieved good performance in facial emotion recognition in autistic children, reducing latency and improving the readiness of supportive responses (35). In the field of social robotics, the integration of artificial intelligence technologies has also been positively evaluated as a structuring tool for promoting social and communication skills (36). Future studies should not only evaluate the effectiveness of currently available technologies, but also monitor how AI-based models can be improved, adapted, and modified over time.

## References

[1] Talantseva OI Romanova RS Shurdova EM Dolgorukova TA Sologub PS Titova OS . The global prevalence of autism spectrum disorder: A three-level meta-analysis. Front Psychiatry. (2023) 14:1071181. doi: 10.3389/fpsyt.2023.1071181, PMID: 36846240 PMC9947250

[2] Wang L Wang B Wu C Wang J Sun M . Autism spectrum disorder: Neurodevelopmental risk factors, biological mechanism, and precision therapy. Int J Mol Sci. (2023) 24:1819. doi: 10.3390/ijms24031819, PMID: 36768153 PMC9915249

[3] He C Miyamoto S Lin Y . Narratives of Chinese kindergarteners with autism spectrum disorder: Comparison with typically developing children. Clin Linguistics Phonetics. (2025), 1–25. doi: 10.1080/02699206.2025.2451968, PMID: 39853168

[4] Walkowiak E . Neurodiversity of the workforce and digital transformation: The case of inclusion of autistic workers at the workplace. Technol Forecasting Soc Change. (2021) 168:120739. doi: 10.1016/j.techfore.2021.120739

[5] Tomczak MT Kulikowski K . Toward an understanding of occupational burnout among employees with autism – the Job Demands-Resources theory perspective. Curr Psychol. (2024) 43:1582–94. doi: 10.1007/s12144-023-04428-0, PMID: 37359683 PMC9958323

[6] Austin RD Pisano GP . Neurodiversity as a competitive advantage. Harvard Business Rev. (2017) 95:96–103.

[7] Hendricks JS Howerton DM . Teaching values, teaching stereotypes: sex education and indoctrination in public schools. U Pa J Const L. (2010) 13:587.

[8] Davies J Melinek R Livesey A Killick E Sam E Romualdez AM . I did what I could to earn some money and be of use”: A qualitative exploration of autistic people’s journeys to career success and fulfilment. Open Sci Framework. (2024), 1–25. doi: 10.31219/osf.io/53h8d, PMID: 39704020 PMC11967104

[9] Tomczak M Wójcikowski M Listewnik P Pankiewicz B Majchrowicz D Jędrzejewska-Szczerska M . Support for employees with ASD in the workplace using a Bluetooth skin resistance sensor—A preliminary study. Sensors. (2018) 18:3530. doi: 10.3390/s18103530, PMID: 30347649 PMC6210705

[10] Bury SM Flower RL Zulla R . Workplace Social Challenges Experienced by Employees on the Autism Spectrum: An International Exploratory Study Examining Employee and Supervisor Perspectives. J Autism Dev Disord. (2021) 51:1614–27. doi: 10.1007/s10803-020-04662-6, PMID: 32809168

[11] Varriale L Briganti P Volpe T Minucci G . Digital technologies for promoting the inclusion of workers with disabilities: A brief investigation. ITM Web Conferences. (2023) 51:3001. doi: 10.1051/itmconf/20235103001

[12] Tomczak MT . Employees with autism spectrum disorders in the digitized work environment: Perspectives for the future. J Disability Policy Stud. (2021) 31:195–205. doi: 10.1177/1044207320919945

[13] Lebene Richmond Soga Y Bolade-Ogunfodun Y Mariani M Nasr R Laker B . Unmasking the other face of flexible working practices: A systematic literature review. J Business Res. (2022) 142:648–62. doi: 10.1016/j.jbusres.2022.01.024

[14] Gal E Selanikyo E Bar-Haim Erez A Katz N . Integration in the vocational world: How does it affect quality of life and subjective well-being of young adults with ASD. Int J Environ Res Public Health. (2015) 12:10820–32. doi: 10.3390/ijerph120910820, PMID: 26404341 PMC4586645

[15] Weber C Krieger B Häne E Yarker J McDowall A . Physical workplace adjustments to support neurodivergent workers: A systematic review. Appl Psychol. (2024) 73:910–62. doi: 10.1111/apps.12431

[16] Scott M Milbourn B Falkmer M Black M Bӧlte S Halladay A . Factors impacting employment for people with autism spectrum disorder: A scoping review. Autism. (2019) 23:869–901. doi: 10.1177/1362361318787789, PMID: 30073870

[17] Migliore A Butterworth J Zalewska A . Trends in vocational rehabilitation services and outcomes of youth with autism: 2006–2010. Rehabil Couns Bull. (2014) 57:80–9. doi: 10.1177/0034355213493930

[18] Burgess S Cimera RE . Employment outcomes of transition-aged adults with autism spectrum disorders: A state of the states report. Am J Intellectual Dev Disabil. (2014) 119:64–83. doi: 10.1352/1944-7558-119.1.64, PMID: 24450322

[19] Seaman RL Cannella-Malone HI . Vocational skills interventions for adults with autism spectrum disorder: A review of the literature. J Dev Phys Disabil. (2016) 28:479–94. doi: 10.1007/s10882-016-9479-z

[20] Costello E Kilbride S Milne Z Clarke P Yilmaz M MacMahon ST . A professional career with autism: Findings from a literature review in the software engineering domain. In: Yilmaz M Clarke P Messnarz R Reiner M , editors. Systems, software and services process improvement, vol. 1442 . Cham, Switzerland: Springer (2021). *EuroSPI 2021. Communications in Computer and Information Science*. doi: 10.1007/978-3-030-85521-5_23

[21] Baldwin S Costley D Warren A . Employment activities and experiences of adults with high-functioning autism and Asperger’s disorder. J Autism Dev Disord. (2014) 44:2440–9. doi: 10.1007/s10803-014-2112-z, PMID: 24715257

[22] De Schipper E Mahdi S de Vries P . Functioning and disability in autism spectrum disorder: A worldwide survey of experts. Autism Res. (2016) 9:959–69. doi: 10.1002/aur.1592, PMID: 26749373 PMC5064728

[23] Walsh L Lydon S Healy O . Employment and vocational skills among individuals with autism spectrum disorder: Predictors, impact, and interventions. Rev J Autism Dev Disord. (2014) 1:266–75. doi: 10.1007/s40489-014-0024-7

[24] Lorenz T Brüning CR Waltz M Fabri M . Not a stranger to the dark: Discrimination against autistic students and employees. Adv Autism. (2021) 7:60–72. doi: 10.1108/AIA-10-2019-0036

[25] Tomczak MT Szulc JM Szczerska M . Inclusive communication model supporting the employment cycle of individuals with autism spectrum disorders. Int J Environ Res Public Health.;. (2021) 18:4696. doi: 10.3390/ijerph18094696, PMID: 33925072 PMC8125785

[26] Zhang M Ding H Naumceska M Zhang Y . Virtual reality technology as an educational and intervention tool for children with autism spectrum disorder: Current perspectives and future directions. Behav Sci. (2022) 12:138. doi: 10.3390/bs12050138, PMID: 35621435 PMC9137951

[27] Basto A. T. O. D. S. Cepellos VM . Autism in organizations: Perceptions and actions for inclusion from the point of view of managers. Cadernos EBAPE.BR. (2023) 21:e2022–0061. doi: 10.1590/1679-395120220061x

[28] Dei C Meregalli Falerni M Cilsal T Ricci M Nunes R Carvalho S . Design and testing of (A)MICO: a multimodal feedback system to facilitate the interaction between cobot and human operator. J Multimodal User Interfaces. (2025) 19:21–36. doi: 10.1007/s12193-024-00444-x

[29] Waardenburg T van Huizen N van Dijk J Magnée M Staal W Teunisse J-P . Design your life: user-initiated design of technology to support independent living of young autistic adults. In: Soares MM Rosenzweig E Marcus A , editors. Design, User Experience, and Usability: Design for Diversity, Well-being, and Social Development, vol. 12780 . Springer, Cham (2021). HCII 2021. Lecture Notes in Computer Science. doi: 10.1007/978-3-030-78224-5_26

[30] Piedade P Neto I Pires AC Prada R Nicolau H . (2023). Partiplay: A participatory game design kit for neurodiverse classrooms, in: Proceedings of the 25th International ACM SIGACCESS Conference on Computers and Accessibility, New York, NY, USA: Association for Computing Machinery (ACM). pp. 1–5.

[31] Kildahl AN Helverschou SB Bakken TL Oddli HW . If we do not look for it, we do not see it”: Clinicians’ experiences and understanding of identifying post-traumatic stress disorder in adults with autism and intellectual disability. J Appl Res Intellectual Disabil. (2020) 33:1119–1132. doi: 10.1111/jar.12734, PMID: 32285568

[32] Beardon L Chown N Cossburn K . First responders and autism. In: Volkmar F , editor. *Encyclopedia of autism* sp*ectrum disorders*. New York, NY: Springer (2018). doi: 10.1007/978-1-4614-6435-8_102159-1

[33] Love AM Railey KS Phelps M Campbell JM Cooley-Cook HA Taylor RL . Preliminary evidence for a training improving first responder knowledge and confidence to work with individuals with Autism. J Intellectual Disabil Offending Behav. (2020) 11:211–9. doi: 10.1108/JIDOB-04-2020-0007

[34] Landowska A Karpus A Zawadzka T Robins B Erol Barkana D Kose H . Automatic emotion recognition in children with autism: A systematic literature review. Sensors. (2022) 22:1649. doi: 10.3390/s22041649, PMID: 35214551 PMC8875834

[35] Talaat FM . Real-time facial emotion recognition system among children with autism based on deep learning and IoT. Neural Computing Appl. (2023) 35:12717–28. doi: 10.1007/s00521-023-08372-9

[36] Santos L Annunziata S Geminiani A Ivani A Giubergia A Garofalo D . Applications of robotics for autism spectrum disorder: A scoping review. Rev J Autism Dev Disord. (2025) 12:455–76. doi: 10.1007/s40489-023-00402-5

